# The Treatment of Acute Maniacal Delirium

**Published:** 1893-12-02

**Authors:** Cecil F. Beadles

**Affiliations:** Assistant Medical Officer, Colney Hatch Asylum


### The Hospital Clinic.

*[The Editor will be glad to receive offers of co-operation and contributions from members of the profession. All letters should be addressed to* The Editor, The Lodge, Porchester Square, London, W.]

### THE TREATMENT OF ACUTE MANIACAL DELIRIUM.

### Means Directed to Relieve the Acute Symptoms and the Subsequent Treatment.

By Cecil F. Beadles, M.R.C.S., L.R.C.P., Assistant Medical Officer, Colney Hatch Asylum.

Acute delirious mania, in the restricted sense of the term, forms a comparatively small percentage of the cases in a pauper lunatic asylum. By this I mean an attack of acute mania coming on suddenly, which is associated with delirium, runs a rapid course to recovery or death, and if recovery takes place does not again occur for a considerable period, or not at all. Such cases more often than not owe their origin to some mental cause, but may follow on one of the acute specifics, a drinking bout, or some other physical condition. This form of insanity has many points of difference from the ordinary acute mania, and while the future is more hopeful, the attack while it lasts is far more severe, and the immediate effects more serious.

But if we use the term so as to include those paroxysms to which epileptics are liable, either before or after a fit, or which may take the place of one, then it is by no means rare, for the number of insane epileptics is very large. They are then of more or less periodic occurrence at longer or shorter intervals, and may exist through many years. Apart from the presence of fits in the epileptic patient, there is little to distinguish between the violent paroxysms while they last, but it is to be noted that the prostration following is usually far less marked, and may be entirely absent in an epileptic. The treatment during an attack in either case is almost identical, and that here referred to may be taken as applying to both. When speaking of treatment, it is important to remember that persons the subject of acute maniacal delirium are generally of a comparatively young age, and that the attack, although it may be prolonged beyond a month, is more often of a few hours or days in duration. Although it is usually impossible to mistake this condition, yet there are times when it is difficult to make a clear diagnosis between acute delirious mania and general paralysis of the insane—a matter of no small importance as affecting the prognosis.

The patient is restless in the extreme, noisy and deluded. He is continually on the move, throwing himself about and striking the walls with his hands, and it may be his head. He sleeps neither day nor night. He shouts and raves and seldom ceases from talking rambling incoherent rubbish, in which delusions play a prominent part.

Our first consideration in such a case is to place the patient in a position where he can injure neither himself nor others. This may be best accomplished by placing the patient in a padded room, where the floor and the walls to a height beyond the ordinary reach are protected, and the window is out of harm's way. If the patient is inclined to be destructive as is so often the case, it may be necessary tc replace the ordinary bedding and clothing by canvas ones. No bedstead or other article of furniture should remain in the room, and the necessary utensils are best fitted into the wall. As these patients are usually of dirty habits somewhat frequent changing of clothing may be required. Whilst the violent state exists there should be as little disturbance as possible. He is better not looked at too frequently, for all unnecessary interference only tends to aggravate the condition. It will be found that darkening the room will often have a quieting influence. On the rarest occasions only need any form of restraint be used. Scarcely once during the whole year is this found necessary at Colney Hatch with its 2,300 patients and its 570 fresh admissions; it is, however, easy to understand how it may be more requisite in private or in institutions where padded rooms do not exist.

Our next care is to procure sleep for the patient if possible, for by this means we not only save the useless expenditure of energy, but, what is even more important, we may succeed in cutting short the attack. It will frequently be found that there have been sleepless nights for some time previous to the onset of a delirious attack, and unless rest can be procured the patient will only exhaust himself the more by his violent exertions, and make the chances of recovery less hopeful.

For such cases as these sulphonal and paraldehyde are practically useless. They are only of use in simple insomnia, where they may be employed for long periods as night draughts in doses of twenty grains and one drachm respectively. For this purpose they answer at times, although personally I have never placed much reliance in them, and have on many occasions known them to completely fail. It is to be remembered that sulphonal is a drug that should be used with caution, as many cases of poisoning have occurred, some of which have proved fatal; but as regards paraldehyde no fatal case is on record. With regard to the action of some of the more recently introduced hypnotics I have had no personal experience, but they appear of little value in maniacal excitement. Chlorobrom, for instance, has failed in this class of cases, although it has been spoken well of in the simple insomnia of melancholia, when it is given in doses of one to one and a half ounce an hour before bedtime. Hypnal (a compound of chloral hydrate 45 per cent. and antipyrin 55 per cent), has been recommended in milder forms of excitement, and in commencing delirium tremens in doses of 15 to 45 grains. Amongst other new drugs of this class are trional and tetronal, which are said to possess advantages over sulphonal owing to their more rapid action. Urethane has been tried, but it possesses no special advantages.

Notwithstanding the many drugs that have been employed from time to time, it invariably comes to our harking back to the older and more tried ones, chloral hydrate and potassium bromide. There is no doubt that these two drugs in combination form the most valuable hypnotic we possess, whether they are used in small doses as an occasional draught for persistent insomnia, or in larger ones to quiet and produce sleep in cases of violent or acute delirious mania. A useful draught is one composed of 20 grains of each, although I prefer as a rule to increase the amount of the bromide and lessen that of the chloral, even when the case is not one of epilepsy. Where there is much excitement it may be necessary to increase the dose to half a drachm of each, which will rarely fail to procure sleep. A mixture which has been somewhat extensively employed of late in the more obstinate cases of insomnia with excitement, is that going by the name of bromidia; its composition as employed at Colney Hatch is as follows: Chloral hydrate and potassium bromide of each gr. xv., tinct. hyoscyamus ♏ x., tinct. canabis indica ♏ v. with water to the ounce, in which doses it is given with a fair amount of success.

Failing to quiet the patient or produce sleep by any of the foregoing drugs, we may be obliged to have recourse to hyoscyamine. This should only be used in exceptional cases where the patient is unusually noisy and violent.

The dose used would depend upon the condition of the patient, and whether he had previously taken the drug. Owing to its power it should be used with great caution. In the first instance a solution containing one-twelfth of a grain might be employed without much risk, increasing to one-fourth of a grain, beyond which it is seldom required to pass, unless the patient has become so tolerant that the dose will not produce the desired effect. I have myself ordered half a grain, and have known one grain to be given, but the requirement of such large doses is very exceptional and should be carefully avoided as not by any means free from danger. It may be impossible to get the patient to swallow the medicine, or, perhaps, after taking it into his mouth he will immediately reject it and then it becomes necessary to give it hypodermically, in which case the dose would be slightly less. In fact it is in those cases that refuse to take anything by the mouth that hyoscyamine proves of use. It is probable that the patient would require to be held by several persons while the injection was being given. In a very short space of time the drug begins to take effect, the patient acts and feels like a drunken man, he staggers in his gait, becomes confused, gradually ceases to talk, loses power over his limbs, lies down and soon falls into a heavy sleep which may last some hours. When he awakes he is more or less confused, but this soon passes off and the patient will usually be found much quieter and better, and frequently to have recovered from his acute attack of mania. If, however, he soon relapses it may be necessary to repeat the dose.

